# *Beauveria bassiana* Lipase A expressed in *Komagataella (Pichia) pastoris* with potential for biodiesel catalysis

**DOI:** 10.3389/fmicb.2015.01083

**Published:** 2015-10-07

**Authors:** Ana C. Vici, Andrezza F. da Cruz, Fernanda D. A. Facchini, Caio C. de Carvalho, Marita G. Pereira, Raquel Fonseca-Maldonado, Richard J. Ward, Benevides C. Pessela, Gloria Fernandez-Lorente, Fernando A. G. Torres, João A. Jorge, Maria L. T. M. Polizeli

**Affiliations:** ^1^Departamento de Bioquímica e Imunologia, Faculdade de Medicina de Ribeirão Preto, Universidade de São Paulo, Ribeirão PretoBrazil; ^2^Departamento de Biologia, Faculdade de Filosofia Ciências e Letras de Ribeirão Preto, Universidade de São Paulo, Ribeirão PretoBrazil; ^3^Departamento de Química, Faculdade de Medicina de Ribeirão Preto, Universidade de São Paulo, Ribeirão PretoBrazil; ^4^Departamento de Biotecnología y Microbiología de Alimentos, Instituto de Investigación en Ciencias de los Alimentos, Consejo Superior de Investigaciones Cientificas, MadridEspaña; ^5^Departamento de Biologia Celular, Instituto de Ciências Biológicas, Universidade de Brasília, BrasíliaBrazil

**Keywords:** lipase, *Beauveria bassiana*, heterologous expression, lipase purification, ethanolysis, biodiesel

## Abstract

Lipases (EC 3.1.1.3) comprise a biotechnologically important group of enzymes because they are able to catalyze both hydrolysis and synthesis reactions, depending on the amount of water in the system. One of the most interesting applications of lipase is in the biofuel industry for biodiesel production by oil and ethanol (or methanol) transesterification. Entomopathogenic fungi, which are potential source of lipases, are still poorly explored in biotechnological processes. The present work reports the heterologous expression and biochemical characterization of a novel *Beauveria bassiana* lipase with potential for biodiesel production. The His-tagged *B. bassiana* lipase A (BbLA) was produced in *Komagataella pastoris* in buffered methanol medium (BMM) induced with 1% methanol at 30°C. Purified BbLA was activated with 0.05% Triton X-100 and presented optimum activity at pH 6.0 and 50°C. *N*-glycosylation of the recombinant BbLA accounts for 31.5% of its molecular weight. Circular dichroism and molecular modeling confirmed a structure composed of α-helix and β-sheet, similar to α/β hydrolases. Immobilized BbLA was able to promote transesterification reactions in fish oil, demonstrating potential for biodiesel production. BbLA was successfully produced in *K. pastoris* and shows potential use for biodiesel production by the ethanolysis reaction.

## Background

Microbial lipases (EC 3.1.1.3) constitute a biotechnological important enzyme group because of their versatile properties and the relative ease of large-scale production compared to animal and vegetable homologues (Singh and Mukhopadhyay, 2012). Lipases belong to the carboxyl ester hydrolase family, which present activity against carboxylic ester bonds of triacylglycerols and act on the organic-aqueous interface releasing fatty acids and glycerol (Jaeger and Reetz, 1998; Bancerz and Ginalska, 2007; Lotti and Alberghina, 2007). These enzymes are able to catalyze both hydrolysis and synthesis reactions of esterification, transesterification, and interesterification, depending on the amount of water in the system (Krishna and Karanth, 2001; Gotor, 2002; Hasan et al., 2009). The different catalytic properties are very important for lipase applications, especially in the food, detergents, fine chemical synthesis, pharmaceutical, cosmetics, and biodiesel industries (Wang et al., 2002; Hasan et al., 2006; Freire and Castilho, 2008). There is therefore a strong interest in the investigation of new sources of lipases or in the enhancement of the production of existing sources.

Research on lipase applications is currently focused on the area of biofuels, since production of biodiesel is a promising substitute for diesel oil. Biodiesel can be produced from renewable resources such as vegetable oils, animal oil, and residual oil from industrial and domestic kitchens. In order to this application, lipases in aqueous-restricted environments and the presence of ethanol or methanol results in a transesterification reaction called alcoholysis, giving rise to fatty acid esters (Iso et al., 2001; Matsumoto et al., 2001; Parawira, 2009).

The use of lipases in biodiesel production is advantageous as compared to alkaline chemical catalysis, because the enzymatic route does not result in saponification reactions and the glycerin and biodiesel recovery is simpler with higher purity. In addition, the catalyst recovery is easy when the lipase is immobilized on inert supports, the energy cost is lower since lipases generally operate under mild temperature conditions, and as a consequence the environmental impact is significantly reduced (Yang et al., 2009; Bisen et al., 2010; Cavalcanti-Oliveira et al., 2011; Batista et al., 2013). However, alcoholysis using lipase is more expensive because of the high cost of the biological catalyst and the lower overall productivity as compared to the alkaline catalysis (Wang et al., 1988; Batista et al., 2013). The current studies on enzyme use are focused on increasing production, reducing costs and making lipases more efficient in transesterification reactions. These goals drive the search for new sources of lipases, as well as their overexpression, immobilization on different supports and modification of known enzymes.

The application of entomopathogenic fungi is mainly in the context of crop pest biocontrol, especially in organic farming. There are only a few reports in the biotechnology area, such as lipase production by *Metarhizium anisopliae* (Silva et al., 2005), *Isaria fumosorosea* (Ali et al., 2009, 2014) and by *Beauveria bassiana* (Mustafa and Kaur, 2010). In these molds, lipases play an important role during the infection of insects, hydrolyzing ester bonds in the lipoproteins, fats and waxes that are abundant in the insect exoskeleton (Ali et al., 2014). Hence, the study of entomopathogenic fungi as a source of lipases for application in catalysis may yield novel enzymes with improved features over existing industrial enzymes.

The genome of *B. bassiana* has been partially sequenced in mid-2012 (Xiao et al., 2012), and several genes encoding proteins with putative lipase function have been annotated, suggesting that this organism might be a promising source of this enzyme. This study reports the cloning and expression of one of these putative lipase genes in *Komagataella pastoris* and evaluation of the potential of the heterologous lipase for biodiesel production.

## Materials and Methods

### Fungi Strain, Culture Medium, Total RNA Extraction and cDNA Preparation

*Beauveria bassiana* CFF74 was grown in Khanna medium (Khanna et al., 1995) supplemented with 1% canola oil and 0.1% peptone at 30°C, 100 rpm for 60 h. Mycelia were harvested and frozen in liquid nitrogen. Total RNA was extracted by the TRIzol^®^(Life technologiesTM) methodology. For this purpose, 1.0 mL of Trizol was used for each 50–100 mg of mycelium. Tubes were homogenized until mycelium was completely dissolved and then, incubated for 5 min at room temperature. Samples were quickly homogenized after the addition of 200 μL of chloroform per 1 mL of Trizol used. Subsequently, samples were incubated at room temperature for 10 min and centrifuged at 12,000 *g*, 4°C for 15 min. The aqueous phase was transferred to another clean tube. A volume of 500 μL of isopropanol was added to the samples. After 3 h at room temperature, samples were centrifuged again at 12,000 *g*, 15 min, 4°C and the pellet was washed with 75% ethanol. Samples were homogenized in vortex and centrifuged at 7,000 *g*, 5 min, 4°C. The supernatant was discarded. After drying for 30 min at room temperature, the pellets were resuspended in 100 mL DEPC-water and incubated for 10 min at 55°C. Total RNA was quantified in NanoDrop spectrophotometer (Thermo Scientific) and analyzed in 1.5% agarose gel, in 1x TAE buffer at 80 V for 2 h. After checking the quality of the samples in the gel, mRNA was used to synthesize the complementary DNA (cDNA) using OligodT_20_ and Invitrogen SuperScript^®^ III Reverse Transcriptase Kit (Life TechnologiesTM) according to the manufacturer’s specifications.

### Cloning and Expression on *K. pastoris*

*Beauveria bassiana* cDNA was used as template for PCR amplification of the gene encoding *B. bassiana* Lipase A *(BblA*) using primers – BblAF, (5′-NNNNNNCTCGAGAAAAGAGAGGCTGAAGCTCTTTCTGCGGACATTGACGG-3) and BblAR, (5’-NNNNNNGAATTCAACATCCATACTCTCAGCAAGAAG CTT-3). The underlined sequences correspond to the *Xho*I and *Eco*RI sites, respectively. The full-length BblA coding sequence was PCR amplified by heating at 95°C for 10 min, followed by 35 cycles of 95°C for 15 s, 52°C for 40 s, 72°C for 3 min and final incubation at 72°C for 7 min. The PCR product was purified and digested with *Xho*I and *Eco*RI and cloned into the expression plasmid pPIC9K (Invitrogen, Nærum, Denmark) to produce the recombinant protein fused to the α-factor secretion signal and to the 6His extension at the N- and C-terminus, respectively (Fonseca-Maldonado et al., 2013). The resulting construct (pPIC9CT-*BblA*) was transformed into electrocompetent DH5α *Escherichia coli*, and isolated plasmid DNA was purified and the nucleotide sequence determined. The BbLA nucleotide sequence was analyzed using the available tools at http://www.expasy.org/.*BblA* (Xiao et al., 2012), and was deposited in the GenBank database under accession number XM_008603978.1.

The pPIC9CT-*Bbla* (5 μg) was linearized with *Mss*I (PmeI) (Thermo Scientific) and used to transform competent cells of the strain *K. pastoris* GS115 by electroporation at 1720 V, 5.5 ms (Ʈ), using an electroporator *Eporator* (Eppedorf^®^, USA). *K. pastoris* GS115/pPIC9CTBbla recombinants were grown on solid Minimal Dextrose (MD) plate medium for 3 days. Ninety-six colonies were transferred to Minimal Methanol Tributyrin plate medium (MMT – 1.34% Yeast Nitrogen Base without amino acids [YNB], 1% tributyrin and 1.5% agar sterile medium added with 1% methanol when the culture medium was at 40°C) and incubated for approximately 3 days at 30°C until halos of tributyrin hydrolysis appeared.

Three colonies that produced halo were inoculated into 100 mL buffered minimal glycerol medium (1.34% YNB; 4.10^-5^% biotin; 100 mM phosphate buffer, pH 6.0; 1% glycerol), and cultured in an orbital shaker (200 rpm) at 30°C. After 16 h, the cells were collected and resuspended in 100 mL buffered methanol-complex medium (BMM – 1.34% YNB; 4.10^-5^% biotin; 100 mM phosphate buffer, pH 6.0) in an Erlenmeyer flask of 1 L, with addition of 1% methanol every 24 h. Culture was incubated for up to 144 h at 30°C and 200 rpm, the culture medium was collected by centrifugation and the production of the recombinant protein was analyzed by SDS–PAGE (Laemmli, 1970) and by quantification of lipase activity. The effect of the induction-time and culture medium composition was evaluated in culture media MM (1:34% YNB, 1% methanol added every 24 h), YPM (1% yeast extract and 2% peptone added 1% methanol added every 24 h) and BMM.

### Protein Purification

Culture supernatants of BbLA-G2 transformant was concentrated and buffer exchanged using Hollow Fiber Cartrige, 50000 MMWC, in QuixStand Benchtop (GE Healthcare Bio-Sciences AB, Uppsala, Sweden) concentration system. Concentrated BbLA was purified by two protocols: (I) BbLA, added 500 mM NaCl, was purified by IMAC-Cu^2+^. Resin was previously equilibrated with 500 mM NaCl in 10 mM phosphate buffer, pH 7.0. Proteins were eluted with 250 mM Imidazol and 500 mM NaCl in 10 mM phosphate buffer, pH 7.0. (II) BbLA in 10 mM phosphate buffer was purified by hydrophobic chromatography on Octyl-sepharose resin. BbLA was eluted with increased Triton X-100 concentrations up to 1%. In both protocols, fractions containing enzyme were dialyzed in 10 mM phosphate buffer. Protein purity was checked using 12% SDS–PAGE, and protein concentration was assayed according to Bradford (1976) using bovine serum albumin as the standard.

### Enzymatic Assays and Enzymatic Characterization

The catalytic activity assay, modified from Moreno-Perez et al. (2014), used a spectrophotometer with a thermostated cell and continuous magnetic stirring (500 rpm) for 2 min. Reactions were initialized by mixing 0.1 mL of the soluble BbLA or its immobilized preparations (suspension) were added to 2.0 mL of the *p*NPB solution (2.0 mM *p*-nitrophenyl butyrate (*p*NPB) in 0.05% Triton X-100 and 25 mM sodium phosphate at pH 7.0) at 40°C. The increase in absorbance at 380 nm (ε = 4.430 M^-1^cm^-1^) resulting from *p*NP release was used to calculate enzymatic activity, given as μmol of *p*NPB hydrolyzed per minute per mL of enzyme (U/mL) under the described conditions. Kinetic parameters were determined for *p*NPB hydrolysis, in the best conditions for BbLA activity. Software GraphPad Prism 6.0 was used to calculate the V_max_ and k_M_ values.

The evaluation of the effect of surfactants on BbLA activity was made with 0.05% Triton X-100 was replaced by 0.05% Tween 20, Tween 80, Tergitol 40, CTAB or SDS. A control without surfactant was performed. Temperature and pH effects were evaluated by a 2^2^ Rotational Central Composite Design. The pH (and encoded) values tested were 4.59 (-1.41), 5.0 (-1), 6.0 (0), 7.0 (+1) and 7.41 (+1.41), and temperature (and encoded) values were 35.9°C (-1.41), 40°C (-1), 50°C (0), 60°C (+1) and 64.1 °C (+1.41). Enzyme activity was evaluated discontinuously, where 50 μL of the reaction was added to 50 μL of supersaturated sodium tetraborate, after 1 and 2 min reaction. *p*NPB hydrolysis was measured at 410 nm (ε = 3.420 M^-1^cm^-1^) in a SpectraMax 96-well plate Spectrophotometer (Molecular Devices). Three central point repetitions were added to verify the analysis reproducibility. A confidence level of 95% was considered in the analysis of the variables effect. A polynomial equation was constructed for response surface analysis, and the Student’s *t*-test was used to verify the statistical significance of the regression coefficients. Statistical software Statistica v.12.0 was used to analyze the experimental data.

### Deglycosylation of Recombinant Protein by Endoglycosidase H

Deglycosylation of purified recombinant BbLA was performed by Endo H_f_ (BioLabs, New England), with modifications to the manufacturer’s protocol. The deglycosylation reaction comprised 45 μL purified BbLA (80 ug protein) added to 5 μL 10X denaturation buffer, mixed and heated at 100°C for 10 min. Thereafter, 10 μL G5 reaction buffer, 0.5 μL of Endo H_f_ and 39.5 μL of MilliQ H_2_0 were added. The reaction was incubated at 37°C for 24 h. Deglycosylation was evaluated using 12% SDS–PAGE.

### CD Spectroscopy

Circular dichroism spectra between 190 and 250 nm (far-UV CD) were measured using a JASCO 810 spectropolarimeter (JASCO, Tokyo, Japan) at a protein concentration of 0.04 mg/mL in 0.1 cm path-length quartz cuvettes in 10 mM phosphate buffer pH 7.0. The mean of four accumulated spectra was measured, from which the spectrum of a buffer blank was subtracted.

### Three-Dimensional Model of the BbLA

The secondary sequence was inferred using the PDBSum platform^1^. Homology modeling method (Deane and Blundell, 2003) was carried out using the I-Tasser (Zhang, 2008)^2^. Based on the X-ray structure of EstA (PDB ID: 1UKC), which was selected as template because an X-ray structure of the more homologue lipase from *B. bassiana* was not available. The primary sequence of lipases (BbLA and EstA) were aligned with highest scoring lipase (*B. bassiana* ARSEF 2860) obtained by BLAST using MUSCLE 3.6 (Edgar, 2004), to recognize the catalytic triad. The model quality structure was evaluated through PROCHECK program (Laskowski et al., 1993). The figures were generated using PyMOL^3^.

### BbLA Immobilization on Duolite A568 and Sepabeads-C18 Resins

The BbLA crude extract was dialyzed and equilibrated in 10 mM phosphate buffer, pH 7.0. A sample of 5 mg protein BbLA solution was added to 1 g of support Duolite A568 (derivative named BbLA-Duolite) or Sepabeads-C18 (derivative named as BbLA-C18). The immobilization mixture was maintained at pH 7.0, overnight, at 25°C. The activity of the immobilized enzyme was measured by the *p*NPB assay.

### Enzymatic Synthesis of Ethyl Esters of Omega-3 Fatty Acids

Ethyl ester synthesis by BbLA was performed as described by Moreno-Perez et al. (2014). First, 0.26 g of dried immobilized lipase was added to the substrate solution containing 0.623 mmol of sardine oil and 6.2 mmol of ethanol dissolved in 4.11 mL cyclohexane. Second, 0.26 g of dry molecular sieves was added to the reaction mixture for the BbLA reaction in the absence of water. The final concentration of sardine oil in the solution was 125 mM, and the reaction was carried out at 25°C. Aliquots of 100 μL were withdrawn every 24 h, and diluted in 300 μL of HPLC mobile phase. BbLA-derivatives used in this experiment were dried using acetone solutions in water with increasing concentrations of acetone up to 100%. Product formation was monitored by HPLC, where the control consisted of the same quantities of reagents, but without the presence of catalyst.

### HPLC Analysis

Reactants and products were analyzed using a reverse-phase column (Ultrabase-C8, 150 mm × 4.6 mm, 5 μm) by RP-HPLC (Spectra Physic SP 100 coupled with a UV detector Spectra Physic SP 8450). Products were eluted at a flow rate of 1.5 mL/min with acetonitrile/water/CH_3_COOH (80:20:0.1 v:v), pH 3. The UV detection was performed at 215 nm. Product yields were calculated from the pure peak areas of known quantities of EPA ethyl ester [retention time (RT) of 9 min] and docosahexaenoic acid (DHA) ethyl ester (RT of 12 min).

### Reproducibility of the Results

All data are the mean of at least three independent experiments.

## Results and Discussion

### Cloning and Expression of *Beauveria bassiana* Lipase A in *Komagataella pastoris*

**FIGURE 1:**
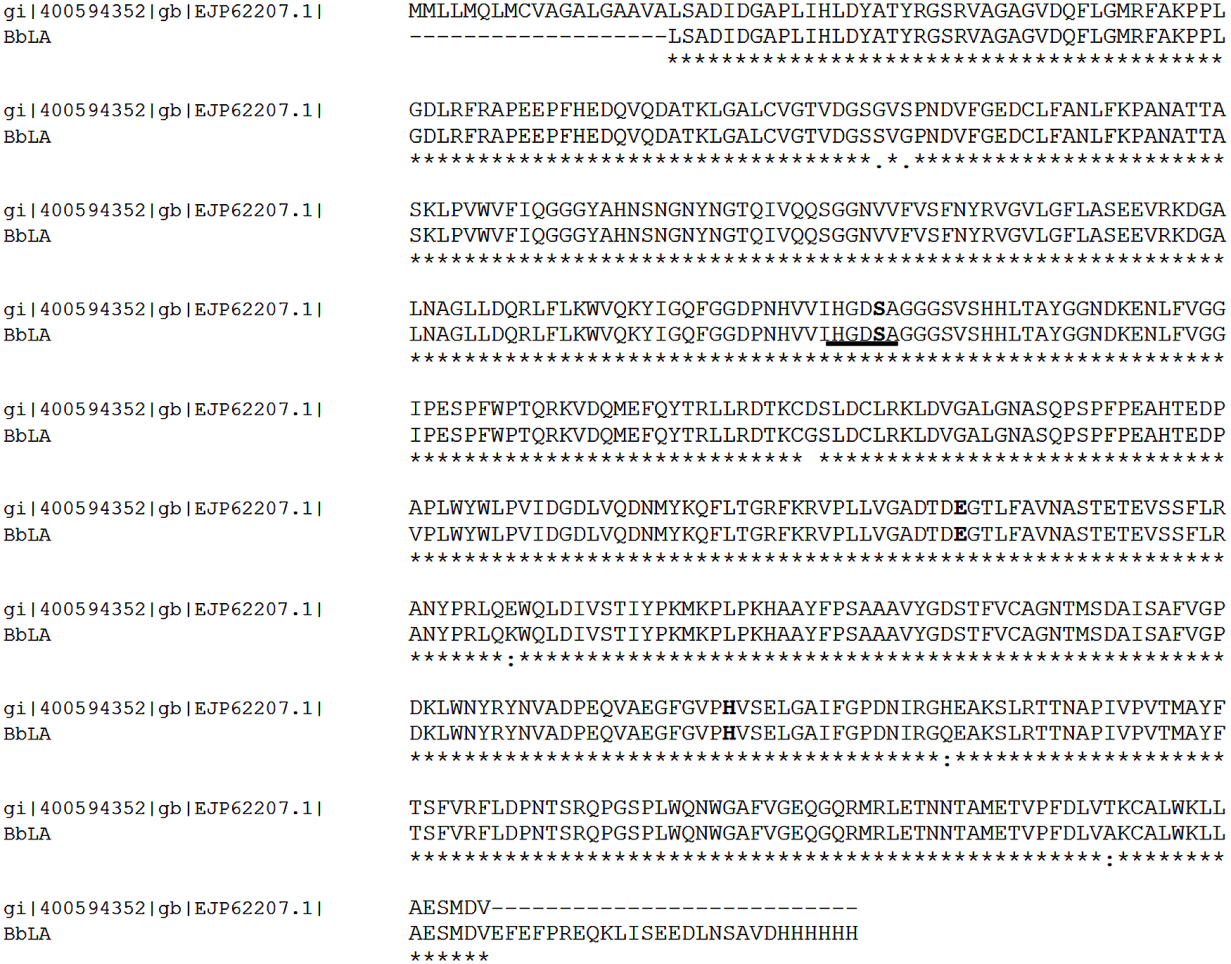
**Amino acid sequence alignment of BbLA with GenBank accession number EJP62207.** The catalytic triad is highlighted in bold and the conserved pentapeptide including the catalytic serine is underlined in black. The *BblA* gene was translated from the nucleotide sequence obtained from the cloned gene. The alignment was generated with Standard Protein BLAST tool.

A putative triacylglycerol lipase gene (GenBank access number EJP62207.1) was chosen from several genes described as lipase or lipase-like in the *B. bassiana* genome. The gene, named *BblA (Beauveria bassiana lipase A*) is 1,641 bp long and encodes a protein with 546 amino acid residues, including a 19-amino acid signal peptide. BbLA has a theoretical molecular weight and *pI* of 59.58 kDa and 5.22, respectively. The *BblA* cDNA without the signal peptide sequence was obtained by RT-PCR from *B. bassiana* cDNA with primers BblAF and BblAR. *BblA* was cloned into the expression vector pPIC9k_CT in-frame with the yeast α-factor secretion signal. The resulting plasmid was sequenced and named pPIC9CT-*BblA*. *BblA* is translated into a 554 amino acids protein (including a His tag at the C-terminus), which has the catalytic triad and the common consensus sequence (G-X-S-X-G) for lipases (**Figure 1**).

**FIGURE 2:**
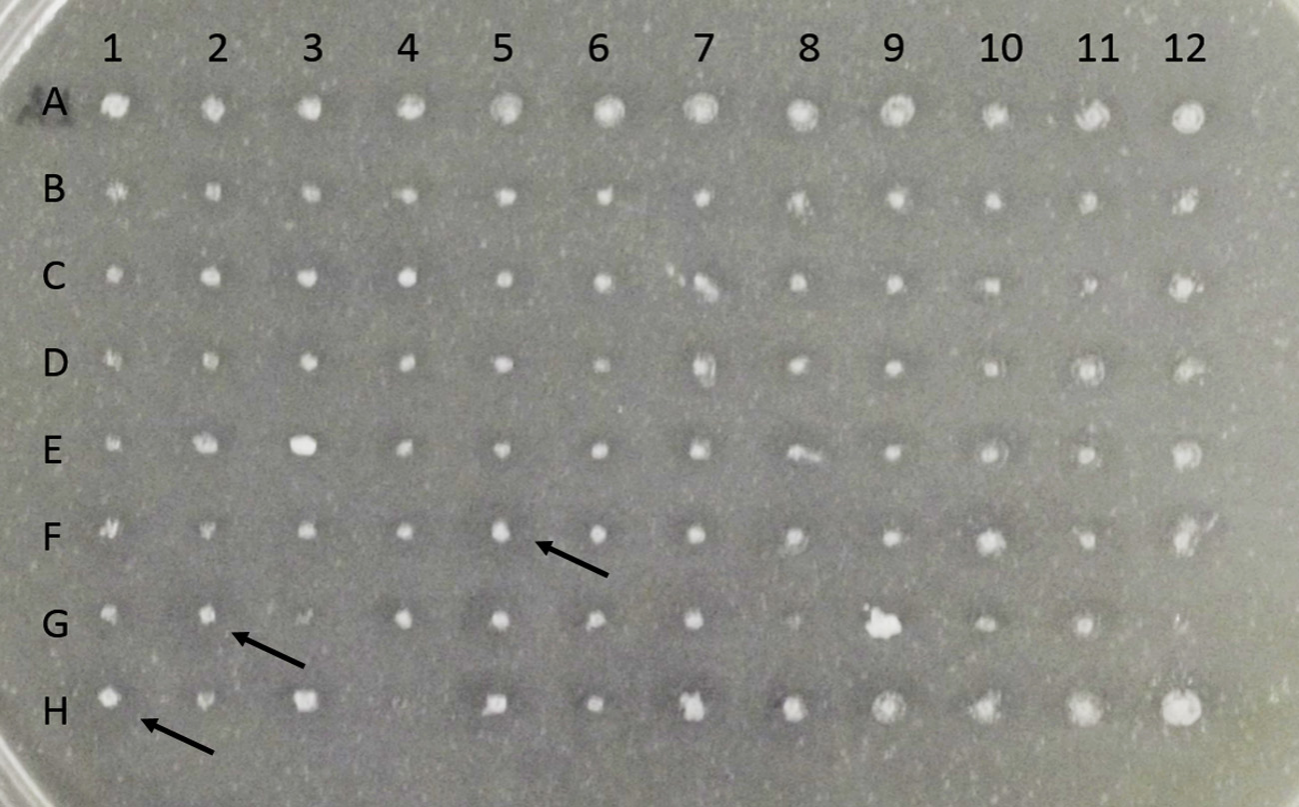
**Screening for expression of BbLA in *Komagataella pastoris* on solid BMM Tributyrin medium.** Colonies were grown at 30° C for 72 h. Arrows indicate the three colonies (F5, G2, and H1) selected for further expression analysis.

Linearized pPIC9CT-*BblA* was used to transform *K. pastoris* GS115. Ninety-six transformants were screened for the presence of hydrolysis halos in MM solid medium containing methanol and tributyrin (**Figure 2**). Three colonies that showed a higher D/d ratio (D/d) were selected. These clones were called BbLA F5, BbLA G2 and BbLA H1.

**FIGURE 3:**
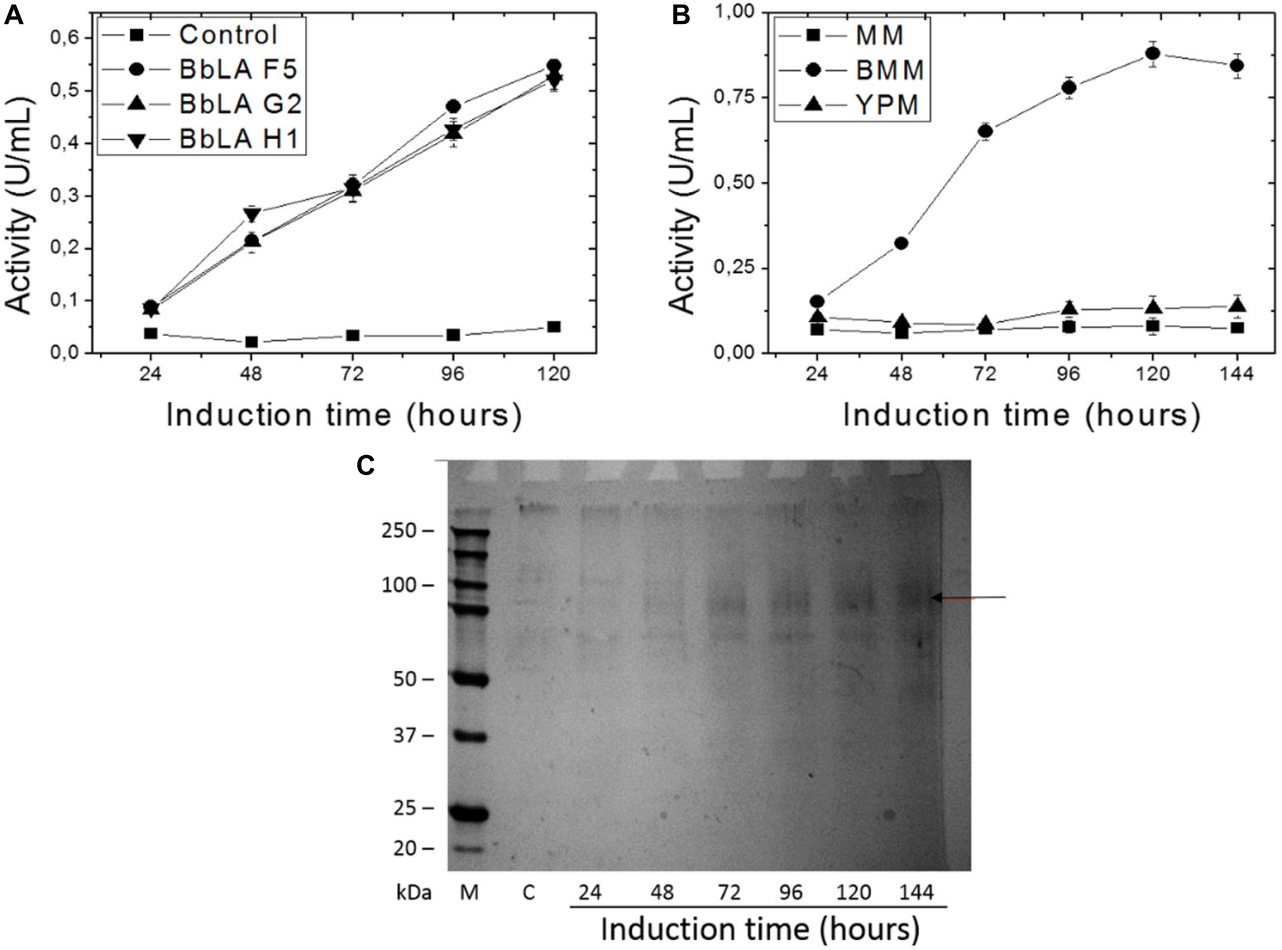
**Recombinant BbLA expression in *K. pastoris*. (A)** Methanol induction of BbLA colonies F5, G2, and H1; **(B)** BbLA expression in different culture medium of the BbLA-G2 colony; **(C)** SDS**–**PAGE electrophoresis of BbLA expressed by BbLA-G2 colony over increasing time intervals on methanol induction. The control was *K. pastoris* transformed with empty vector. M, molecular weight marker; C, control. Cultures were incubated at 30°C, with 200 rpm. The arrow indicates the purified lipase.

After 120 h induction with 1% methanol in liquid medium, the culture supernatants from the three clones showed similar lipase activity (**Figure 3A**). Transformant BbLA G2 was chosen for further studies because it showed the highest ratio D/d (**Figure 2**). BbLA showed better lipase expression in BMM medium after 120 h of methanol induction, demonstrating that buffering the culture medium and long induction times favor protein expression (**Figures 3B,C**).

### Purification and Characterization of BbLA

**FIGURE 4:**
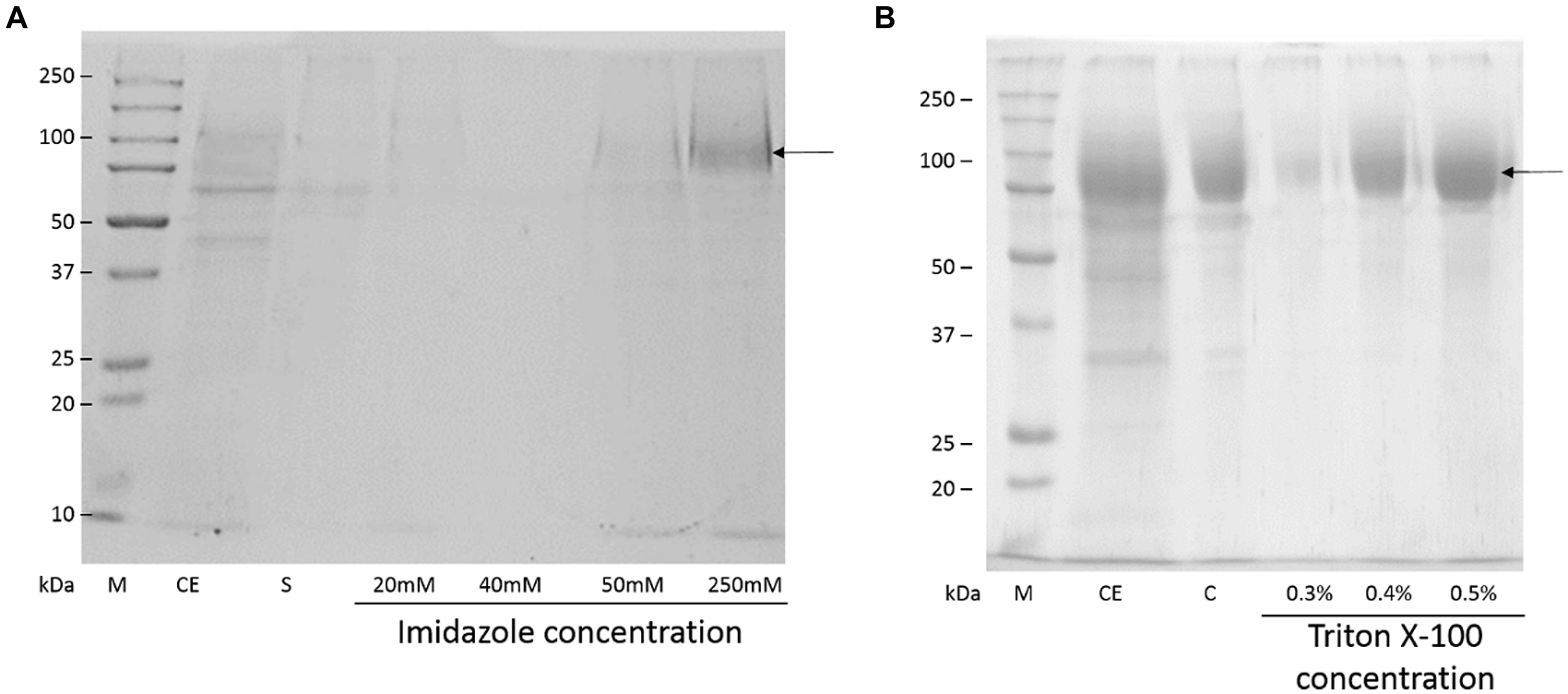
**Sodium dodecyl sulfate–polyacrylamide gel electrophoresis electrophoresis of BbLA purification. (A)** Purification in IMAC – Cu^2+^. **(B)** Purification on Octyl-sepharose. M, molecular weight marker; CD, crude extract of BbLA; S, IMAC supernatant; C, concentrated BbLA. The arrows indicate the band corresponding to BbLA. See Methods section for further experimental details.

*Beauveria bassiana* lipase A was purified by two protocols. The first protocol employed IMAC-Cu^2+^, which exploited the addition of a His-tag at the N-terminal of BbLA. The second protocol involved hydrophobic interaction chromatography using Octyl-sepharose at low ionic strength, which exploited the fact that BbLA is a lipase and therefore has a strongly hydrophobic substrate binding region. Both these protocols resulted in purified BbLA (**Figure 4**). IMAC-Cu^2+^ showed a slightly higher purification factor, while the Octyl-sepharose chromatography showed higher recovery (**Table 1**). Therefore, the sample obtained from Octyl-sepharose purification was used for enzymatic characterization assays.

**Table 1:** *Beauveria bassiana* lipase A purification by immobilized metal (Cu^2+^) affinity chromatography (IMAC) and hydrophobic interaction (Octyl-sepharose) chromatography.

Sample	Specific activity (U/mg)	Purification factor	Recovery (%)
Crude extract	8.59	1	100
IMAC – Cu^2+^	131.50	15.30	39.13
Octyl-Sepharose	119.18	13.88	75.58

*Beauveria bassiana* lipase A was activated by Triton X-100, Tween 20, Tween 80, Tergitol and CTAB, but was completely inactivated by SDS. The best condition for BbLA activation was with 0.05% Triton X-100 (Supplementary Figure S1).

**FIGURE 5:**
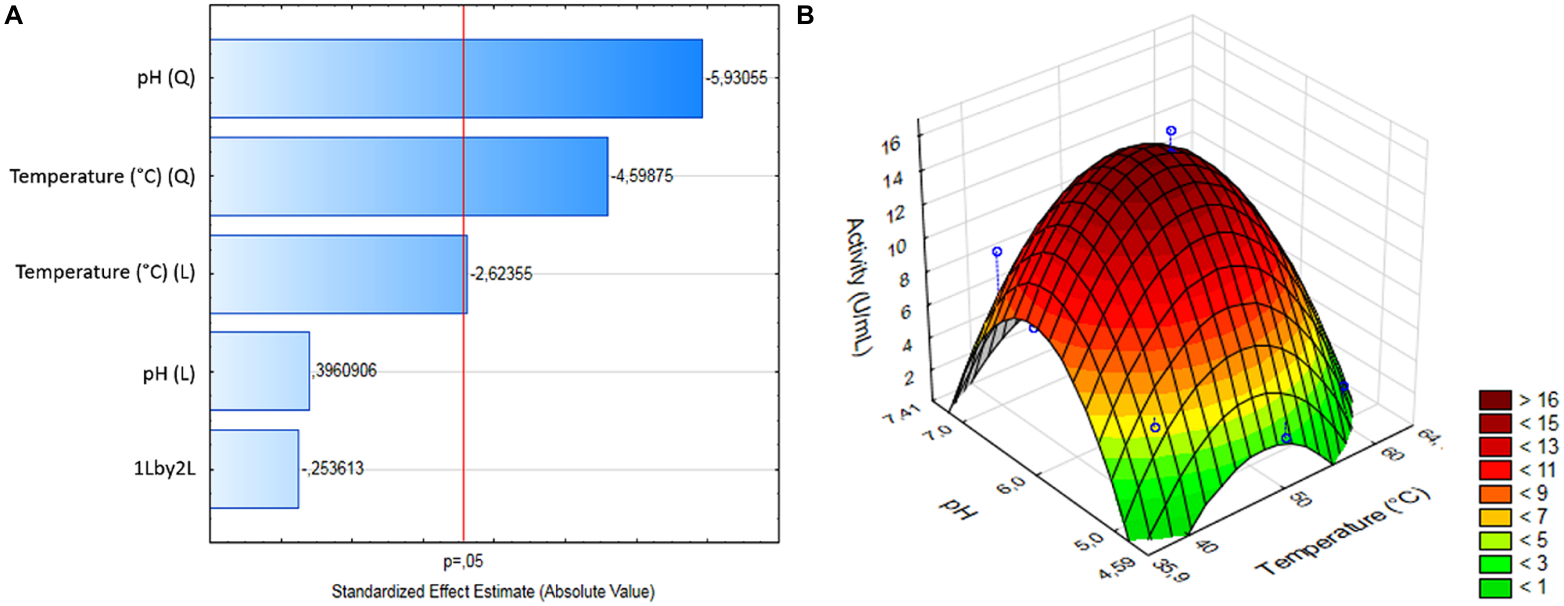
**Experimental design for assessing pH and temperature effects on BbLA activity. (A)** Pareto plot for CCDR. **(B)** Surface response for pH and temperature variables. The analysis was performed by Statistica 12.0 software.

A CCRD was used to analyze the optimal temperature and pH for BbLA reaction. For BbLA activity, the two variables showed a linear effect and temperature showed a quadratic effect (**Figure 5A**). A reduced second order model for BbLA activity is described by equation 1.

(1) $$Activity(U/mL)=15.96-2.43(T)-5.09{\left(T\right)}^{2}-6.56{\left(pH\right)}^{2}\;$$

where, T and pH are encoded temperature and pH values, respectively.

**Table 2** shows ANOVA results from reduced models generated. The determination coefficient was 0.9112. *F*_calculated_ value was higher than *F*_listed_ (**Table 2**). Therefore, this model was considered predictive and statistically significant at 95% confidence level. This result was analyzed by response surface methodology, and generated a 3D surface graph (**Figure 5B**). These results led to the estimation of ideal temperature and pH reaction conditions for BbLA, which were 50°C and pH 6.0, corresponding to the central points assayed.

**Table 2:** Central composite rotational design ANOVA for pH and temperature effects on the BbLA activity.

Variation source	Sum of square (SS)	Degree of freedom (DF)	Mean of Square (MS)	*F*_calculated_ MS_R_/MS_R_	*F*_listed_ *F*_95%,DFR_, _DFr_
Regression (R)	354.17	3	118.057	23.898	4.35
Residue (r)	34.59	7	4.94		
Total (T)	388.76	10			

*R^2^ = 0.9112.*

The K_M_, V_max_, k_cat_ and K_cat_/K_M_ of BbLA recombinant against *p*NPB, pH 6.0 at 50°C, were 0.5546 μM, 85.67 μmols.min^-1^.mg^-1^, 7.139 × 10^4^.s^-1^, and 1.29 × 10^11^.M^-1^.s^-1^, respectively (**Table 3**). Hill coefficient was higher than 1.0, which suggested that BbLA can be an allosteric enzyme. Allosteric lipase modulation is not common, but it is described as possible and biologically relevant (Kohler and Wunsch, 2007).

**Table 3:** *Beauveria bassiana* lipase A kinetic parameters obtained using *p*NPB as substrate in 25 mM phosphate buffer, pH 6.0, after incubation at 50°C, for 2 min.

V_max_ (μmol.min^-1^.mg^-1^)	K_M_ (μM)	K_cat_ (s^-1^)	K_cat_/K_M_ (M^-^1.s^-1^)	*n*
85.67	0.5546	7.139 × 10^4^	1.29 × 10^11^	1.46

*GraphPad Prism 6.0 was used to calculate V_*max*_, K_*M*_ and n. n, Hill coefficient.*

### BbLA Deglycosylation

**FIGURE 6:**
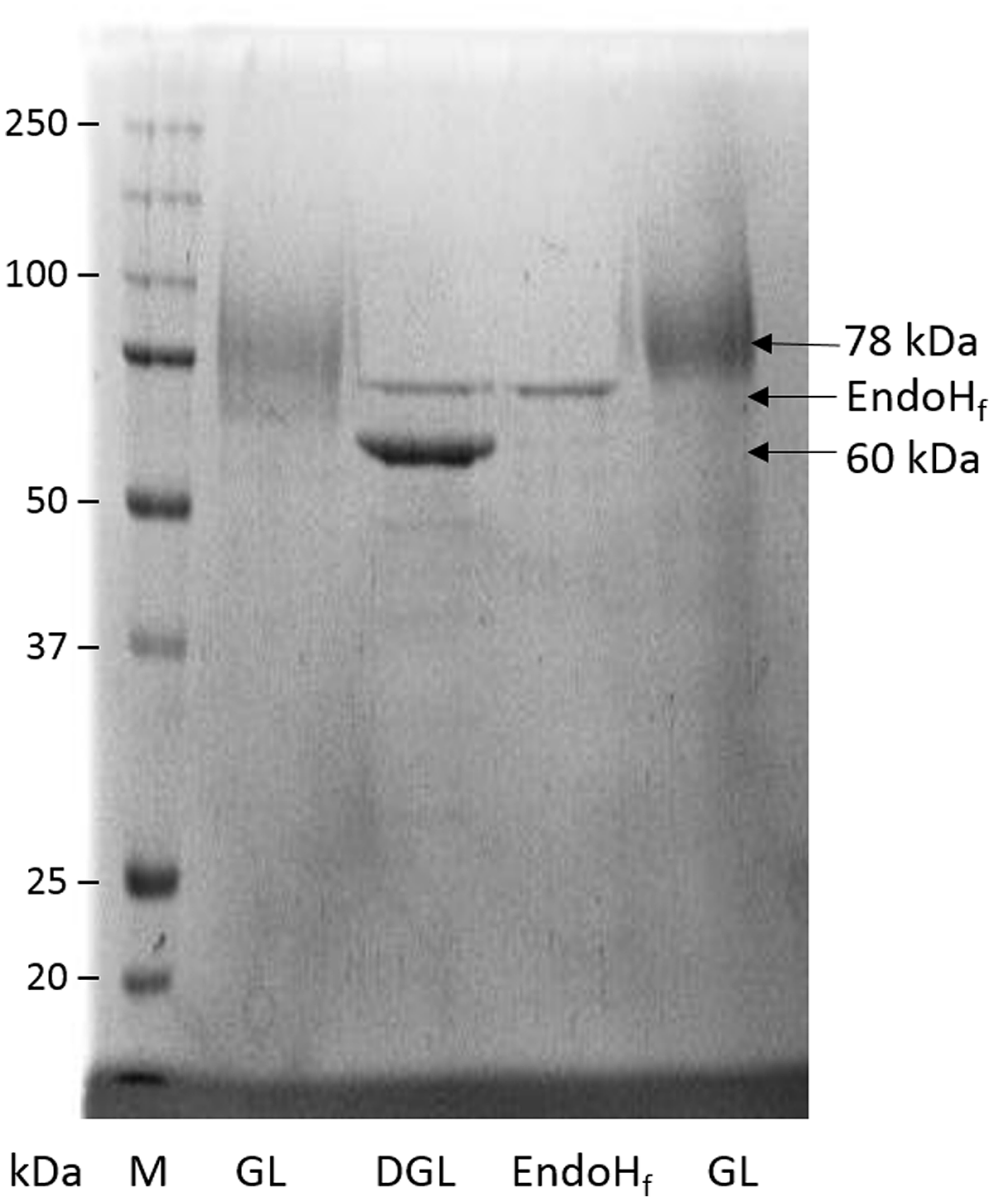
***Beauveria bassiana* lipase A glycosylation analyses by SDS–PAGE.** M, molecular weight marker; GL, glycosylated BbLA; DGL, BbLA treated with endoglycosidase H_f_ (EndoH_f_); EndoH_f_, EndoH_f_ control. See Section “Materials and Methods” for further experimental details.

*Beauveria bassiana* lipase A presents no potential site for *O*-glycosylation, but includes six potential *N-*glycosylation sites. Purified BbLA showed a diffuse band in SDS–PAGE centered around 78 kDa, which after treatment with EndoHf, an endoglucanase that cleaves *N*-glycosylated oligosaccharides, shifts to 60 kDa (**Figure 6**). Assuming that these forms represent the glycosylated and deglycosylated forms, respectively, then the saccharide content of the glycosylated BbLA is approximately 30%.

### Molecular Modeling

**FIGURE 7:**
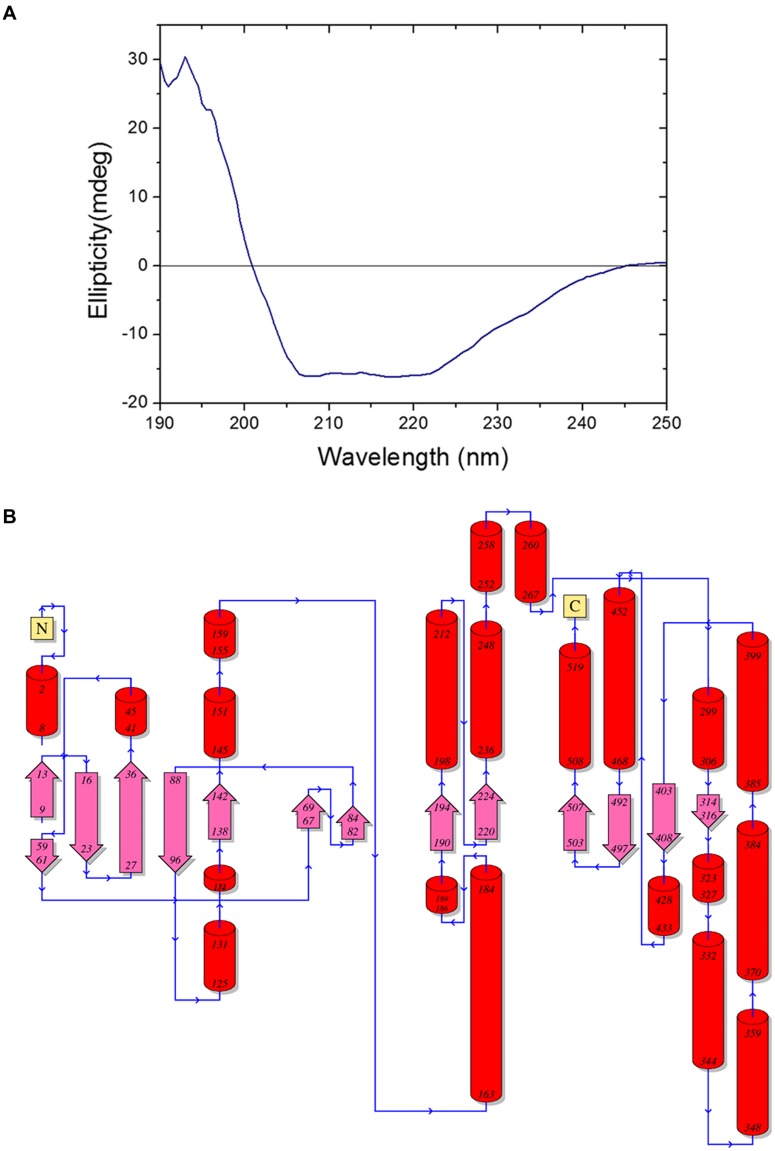
***Beauveria bassiana* lipase A secondary structures. (A)** Far-UV circular dichroism spectrum of BbLA. **(B)** Secondary structures of *Beauveria bassiana* Lipase A. The model was inferred by PDBSum (http://www.ebi.ac.uk/pdbsum/) (Laskowski et al., 1993) using the amino acid sequence. The numbers are regarding residues positions, red cylinders are α-helices and pink arrow are β-strands. N, N-terminal and C, C-terminal.

The far-UV circular dichroism spectrum of purified BbLA at pH 7.0 presents a maximum at 194 nm and a minimum at 207 and 220 nm, which are spectral features typical of proteins rich in α-helix (**Figure 7A**). Nevertheless, the spectral feature generated is not characteristic of proteins exclusively formed by α-helices.

**FIGURE 8:**
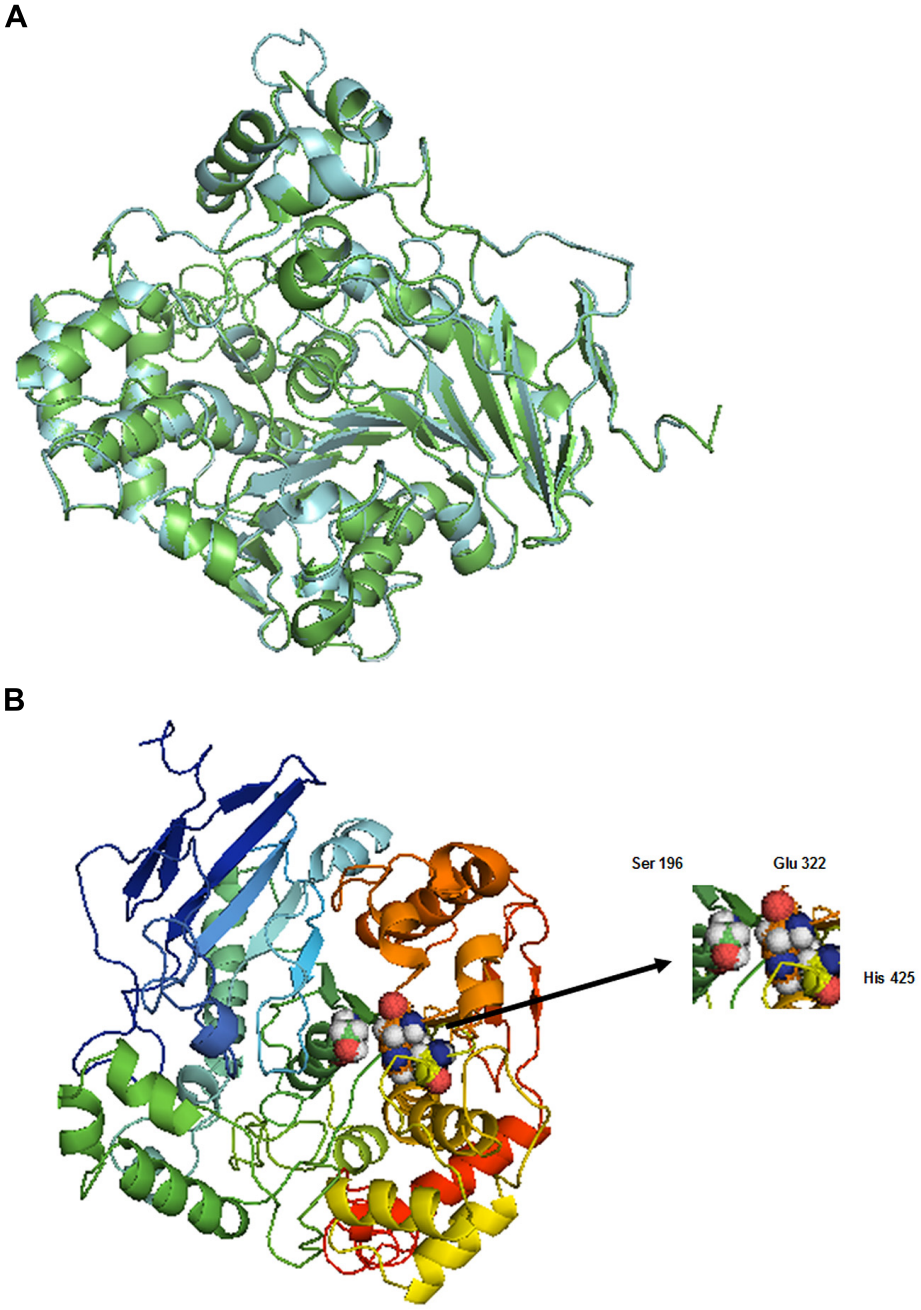
***Beauveria bassiana* lipase A molecular modeling. (A)** Superposition of structures from BbLA (blue) and EstA (green). The figure was made by I-TASSER (Zhang, 2008) using a TM-align structural alignment program. **(B)** 3D model of BbLA showing the catalytic triad (Ser, Glu, and His). The ribbon is colored with a rainbow spectrum from N-terminus (blue) to C-terminus (red).

*Aspergillus niger* esterase (EstA, PDB code Q6ED33) (Bourne et al., 2004), shares 55.56% identity with BbLA and was used as a template for structural modeling of the BbLA on the I-TASSER platform. The secondary structure topology is showed in **Figure 7B** for the sequence length of 554 amino acids, where 61 residues are found in beta strands (forming β-sheets), 153 in α-helices, 14 in 3-10 helix and 326 in other secondary structure motifs such as beta hairpins, beta bulges, beta turns, and gamma turns. The model of the enzyme is a monomer presenting a α/β fold comprised of four central stranded β sheet flanked by 22 α-helices. The superposition between BbLA and EstA structures (**Figure 8A**) generates an RMSD score of 0.37. The catalytic triad is formed by Ser-Glu-His (**Figure 8B**).

### BbLA Immobilization and Application of BbLA-Derivatives in Fish Oil Ethanolysis

*Beauveria bassiana* lipase A was immobilized on Duolite and C18-Sep by reversible anionic and hydrophobic interaction, respectively. Derivatives generated from this immobilization were named BbLA-Duolite and BbLA-C18, and had 8.65 and 12.35 U/g of support, respectively. Duolite and C18-Sep immobilized 83 and 98% of the BbLA present in the crude extract. BbLA-Duolite and BbLA-C18 were dried and added to a reaction medium free of water, in order to favor the transesterification reaction.

Eicosapentaenoic and DHA acids are abundant in sardine oil, and fatty acid ester formation after 24 h reaction revealed BbLA-C18 produced 7.35-fold more EPA-ester and 13.61-fold more DHA-ester than produced by BbLA-Duolite after 48 h reaction. However, BbLA-Duolite was more selective than BbLA-C18 (**Table 4**).

**Table 4:** Sardine oil Ethanolysis catalyzed by Duolite-BbLA and BbLA-C18 derivatives in the presence of cyclohexane.

Derivative	Selectivity (EPA/DHA)	Ethanolysis (%)	
		EPA	DHA
BbLA-Duolite^a^	5.57	0.66	0.18
BbLA-C18^b^	2.98	4.85	2.45

*The reaction occurred at 25°C, 100 rpm. ^a^48 h of reaction. ^b^24 h of reaction.*

## Discussion

*BblA* is a *B. bassiana* putative triacylglycerol lipase gene. This gene encodes a protein with 546 amino acid residues, 59.58 kDa and *pI* 5.22. BbLA protein has the catalytic triad and the common consensus sequence (G-X-S-X-G) for lipases. This sequence was the only consensus sequence identified in studies on non-redundant sequences of lipases deposited in the public data banks. The serine residue present in this pentapeptide is the nucleophilic amino acid present in the catalytic triad (Jaeger et al., 1994, 1999; Schrag and Cygler, 1997; Lotti and Alberghina, 2007).

BbLA F5, BbLA G2 and BbLA H1 clones presented similar lipase activity. BbLA G2 showed better lipase expression in BMM medium after 120 h of methanol induction. In comparison, *Thermomyces lanuginosus* lipase shows improved expression when grown in BMMY medium (similar to BMM, but including yeast extract) pH 7.0, with 1.2% methanol added every 24 h, and 144 h induction (Fang et al., 2014). These results corroborate those obtained in this work and demonstrate the importance of controlling the pH and induction over relatively long periods.

*Beauveria bassiana* lipase A was purified by affinity chromatography (IMAC-Cu^2+^) and hydrophobic interaction chromatography (Octyl-sepharose). The BbLA recovery after IMAC purification (39.13%) was superior to that observed for GCLI (13%) and GCLII (14%) for IMAC purification of *Geotrichum candidum* lipase expressed in *Saccharomyces cerevisiae* (Bertolini et al., 1995). However, it was lower than the recovery obtained for *Bacillus licheniformis* lipase (64%) also fused to a His-tag and expressed in *Escherichia coli* (Madan and Mishra, 2009). BbLA purified with Octyl-Sepharose had 75.58% recovery. Under low ionic strength conditions, only proteins with large hydrophobic sites, such as lipases, adsorb to hydrophobic resins (Palomo et al., 2002, 2006; Mateo et al., 2007). Indeed, hydrophobic interaction chromatography is often used as one of the steps for lipase purification, such as lipases produced by *Pseudomonas aeruginosa* (Bertolini et al., 1995) e *Staphylococcus warneri* (Volpato et al., 2011).

Surfactants activation is common to lipases. Triton X-100, Tween 20, Tween 80, Tergitol and CTAB activated BbLA. While, SDS inactivated it. These results are similar to those previously reported in the literature, such as those obtained for *Thermosyntropha lipolytica* lipases A and B, which are activated by sodium cholate, CTAB, Triton X-100, Tween 20, Tween 80 and SDS (Salameh and Wiegel, 2010). The lipase from *Thermomyces lanuginosus* was also activated by all the surfactants used, included SDS (Mogensen et al., 2005). On the other hand, *Penicillium chrysogenum* lipase was inhibited by almost all detergents used, including SDS, Triton X-100 and Tween 20, the only exception being cholic acid (Bancerz et al., 2005).

Central composite rotational design and response surface showed that the best temperature and pH reaction were 50°C and pH 6.0, respectively. Similarly to the results presented in this work, the optimum conditions obtained for *Penicillium verrucosum* lipase activity (Kempka et al., 2008) were 44°C and pH 7.0 and 40°C and pH 5.0 for *Penicillium* sp. lipase (Wolski et al., 2009).

*Beauveria bassiana* lipase A expressed in *K. pastoris* is approximately 30% *N*-glycosylated. The glycosylation of BbLA was higher than that observed for the *Y. lipolytica* Lip2, expressed in *K. pastoris*, which has 12% of its molecular weight corresponding to glycosylation (Wang et al., 2012). However, not all proteins expressed by *K. pastoris* are glycosylated. For example, two RCLs expressed in this system showed no evidence for glycosylation after treatment with EndoH (Yu et al., 2009).

*Beauveria bassiana* lipase A far-UV circular dichroism spectrum presented features typical of proteins rich in α-helix. But BbLA is not exclusively formed by α-helix, because the secondary structure topology showed that 61 amino acids residues would be in the β-sheet configuration. A similar structure is observed for other lipase enzymes from mammalian and bacterial origin, where the number of α-helices and β-sheet differ from one species to another (Messaoudi et al., 2011).

*Aspergillus niger* esterase A (Bourne et al., 2004) was used as a template for structural modeling of the BbLA. Esterases (EC 3.1.1.1) and lipases (EC 3.1.1.3) can be differentiated on the basis of their substrate specificity and by comparison of sequence-related structural features. In contrast to esterases, lipases display a significant difference in the distribution of hydrophobic amino acid residues in the vicinity of their active sites (Chahinian and Sarda, 2009). The catalytic triad formed by Ser-Glu-His is the same as that in the *Geotrichum candidum* lipase, where glutamic acid replacing the usual aspartate (Schrag et al., 1991).

The three-dimensional model of the BbLA has proportionally more α-helix than β-sheet, but the two secondary structures are present. BbLA circular dichroism and molecular model are consistent with structures of lipases and esterases, because showed similar structure to α/β hydrolase. Despite the validation of structure through Ramachandran plot have presented less than 90% of residues in most favored regions. The predicted model is consistent with other lipases structures from eukaryotic and prokaryotic organisms (Kobayashi et al., 2002; Maraite et al., 2013; Bouchaala et al., 2015).

*Beauveria bassiana* lipase A was immobilized on Duolite and C18-Sep. BbLA-derivatives were assayed in the fish oil ethanolysis to produce biodiesel. BbLA-C18 produced more fatty acid ethyl esters than BbLA-Duolite after 48 h reaction. Nevertheless, BbLA-Duolite was more selective for EPA-ester production than BbLA-C18. For enzymatic biodiesel production, the final yield is the most relevant factor to be evaluated; however the selectivity can be used as the basis for development of enzymatic cocktails of lipases with different specificities, according to the type of oil used.

Lipases from different sources, even when immobilized on the same type of support, may present different selectivity. In a previous ethanolysis study [49], the authors obtained different degrees of selectivity (EPA/DHA) for CALB (3.0), TLL (29.0) and RML (13.0), immobilized on C18-Sep. Catalyst selectivity was influenced by the nature of the support used for immobilization, where TLL-C18 was more specific than TLL-Duolite and the selectivity were 29 and 12, respectively (Moreno-Perez et al., 2014). It is noteworthy that the TLL also showed differences in selectivity when immobilized in the same supports used for BbLA.

Biodiesel production by BbLA-C18 may be improved through the study of several factors, such as increasing the amount of enzyme on the support, changing the organic solvent used, altering the temperature and the efficiency of transesterification on other oils. The present results demonstrate that the search for new enzyme sources together with the modulation of the activity by enzyme immobilization are valuable strategies for the development of biotechnological routes for biofuels production.

## Conclusion

A *B. bassiana* Lipase A was expressed as a heterologous protein in *K. pastoris*, and the biochemical characteristics of the BbLA show potential for biodiesel production. The differential immobilization, by anionic and hydrophobic interaction, can generate products with different proportions of EPA and DHA esters, at mild temperatures, reducing the energy costs for biodiesel production.

## Author Contributions

This work is part of the doctorate thesis of ACV and she is the main author. AC, RF-M, RW, and FT had collaboration in part of the experiments with molecular biology and fungal expression (enzyme assays optimum temperatures and heterologous protein expression). RW performed the circular dichroism. CC performed the molecular modeling BbLA and its analysis. BP, GF-L, FF and MGP had collaboration in the immobilization and application of enzyme. JJ designed some experiments; MLTMP contributed with the experimental design and the final manuscript.

## Supplementary Material

The Supplementary Material for this article can be found online at: http://journal.frontiersin.org/article/10.3389/fmicb.2015.01083

## Conflict of Interest Statement

The authors declare that the research was conducted in the absence of any commercial or financial relationships that could be construed as a potential conflict of interest.

## Footnotes

1 http://www.ebi.ac.uk/pdbsum/

2 http://zhanglab.ccmb.med.umich.edu/I-TASSER/

3 http://www.pymol.org

## Abbreviations

BbLA: *Beauveria bassiana* Lipase A expressed in *Komagataella pastoris*
BMM: buffered methanol medium
bp: basis pair
BMMY: buffered methanol medium plus yeast
C18-Sep: Octadecyl-Sepabeads
CALB: *C. antarctica* lipase B
CCRD: central composite rotational design
CTAB: cetyl trimethylammonium bromide
D/d: diameter of the halo/diameter of colony
DEPC: diethylpyrocarbonate
DHA: docosahexanoic acid
EPA: eicosapentaenoic acid
EstA: *Aspergillus niger* esterase A
Far-UV CD: far ultraviolet Circular Dichroism
GCLI: *Geotrichum candidum* lipase I
GCLII: *Geotrichum candidum* lipase II
GS115: strain of *K. pastoris* used in this work
IMAC-Cu^2+^: immobilized metal (Cu^2+^) affinity chromatography
k_cat_: Turnover number
K_M_: Michaelis constant
MM: minimal medium
PDB: protein data base
*pI*: isoelectric point
*p*NP: *p*-nitrophenol
*p*NPB: *p*-nitrophenyl butirate
pPIC9k_CT: vector used in this work
RCLs: *R. chinensis* lipases
RML: *R. miehei* lipase
RT-PCR: real time PCR
SDS: sodium dodecyl sulfate
SDS–PAGE: sodium dodecyl sulfate–polyacrylamide gel electrophoresis
TLL: *T. lanuginosa* lipase
V_max_: maximum reaction rate
YNB: yeast nitrogen base
YPM: yeast peptone medium.
