# Supplementary material for: Beauveria bassiana Lipase A expressed in Komagataella (Pichia) pastoris with potential for biodiesel catalysis
Source: Front Microbiol. 2015 Oct 7;6:1083. doi: 10.3389/fmicb.2015.01083 (PMC4595793; doi:10.3389/fmicb.2015.01083)
Supplement: Supplementary file 1 [file Data_Sheet_1.DOCX]

## ***Supplementary Material***

***Beauveria bassiana* Lipase A expressed in *Komagataella (Pichia) pastoris* with potential for biodiesel catalysis**

Ana Claudia Vici, Andrezza Furquim da Cruz, Fernanda Dell Antonio Facchini, Caio Cesar de Carvalho, Marita Gimenez Pereira, Raquel Fonseca-Maldonado, Richard John Ward, Benevides Costa Pessela, Gloria Fernandez-Lorente, Fernando Araripe Gonçalves Torres, João Atílio Jorge, Maria de Lourdes Teixeira Moraes Polizeli*

* **Correspondence:** Dr Maria de Lourdes Teixeira de Moraes Polizeli: polizeli@ffclrp.usp.br

**1. Supplementary Figures**

**
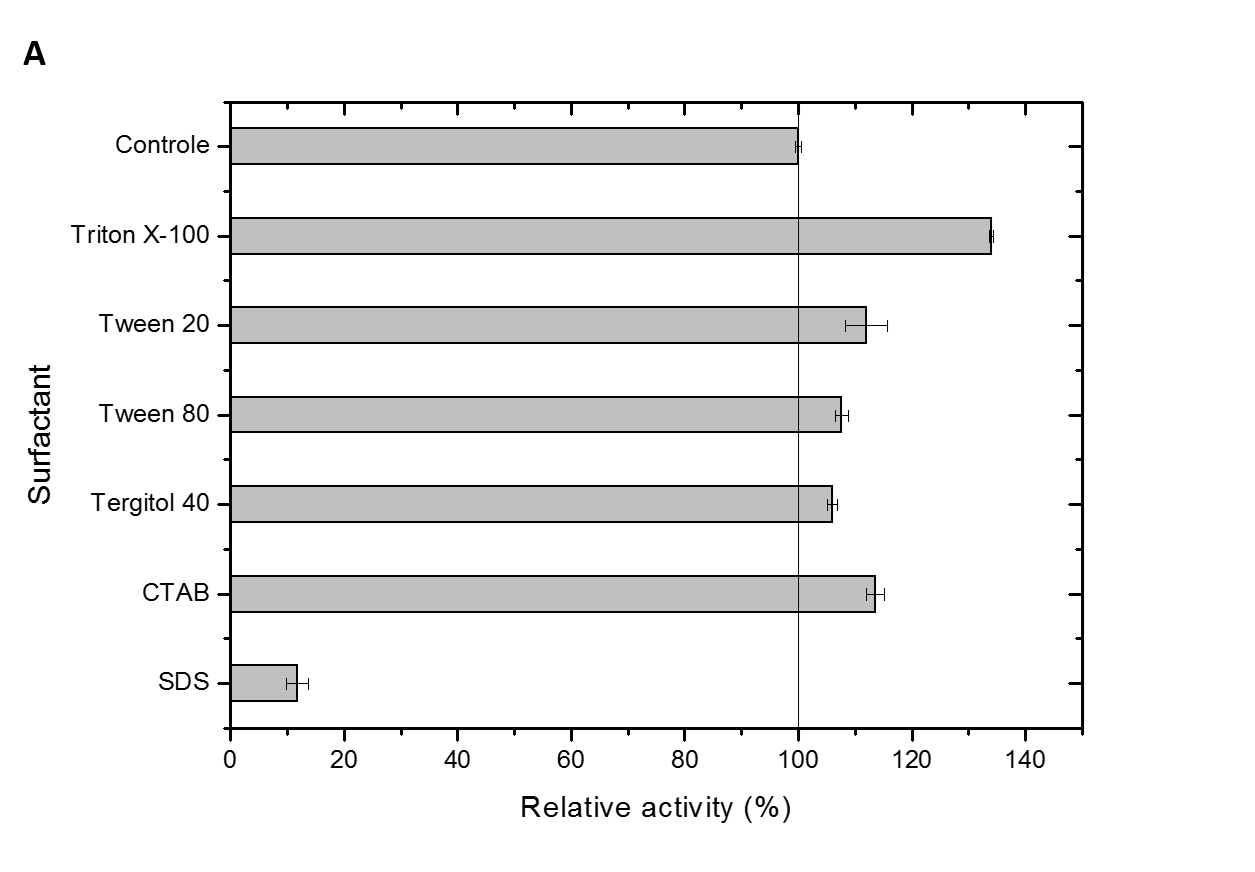
**
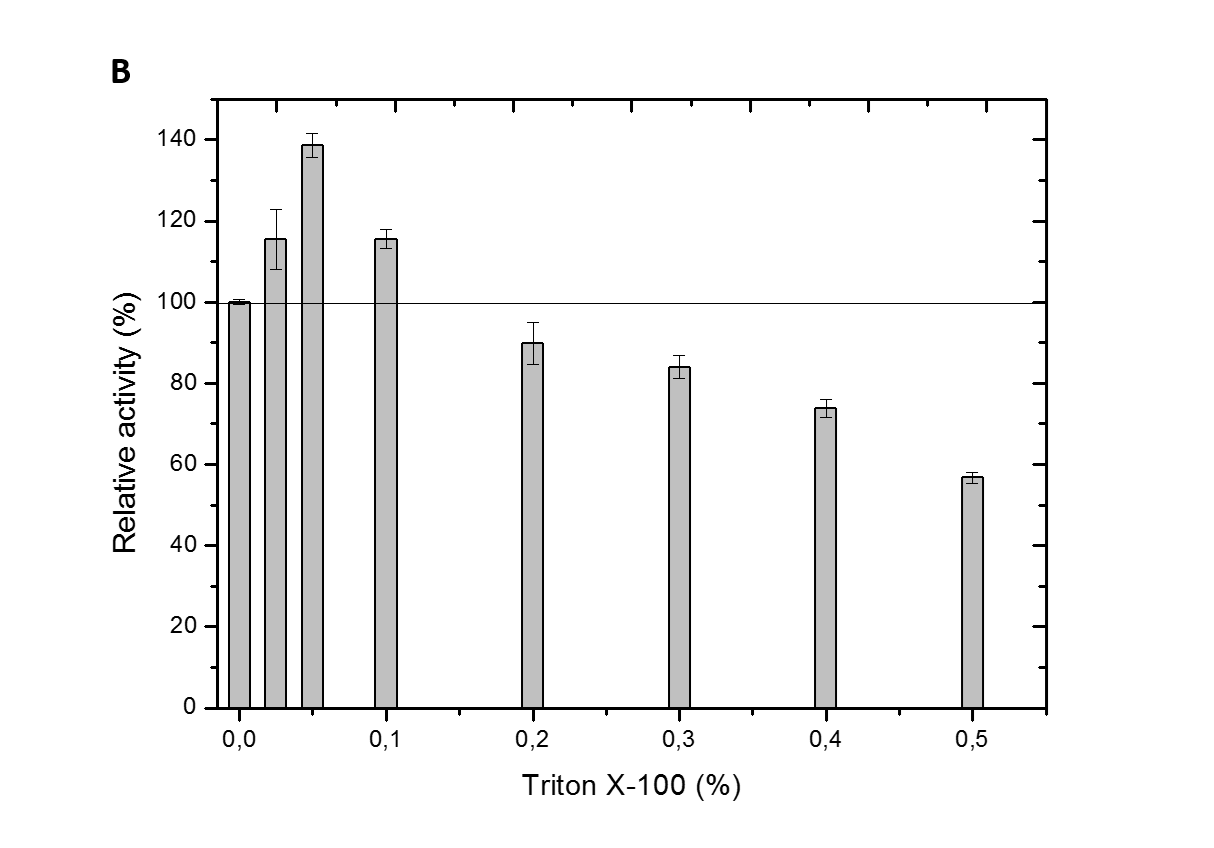


**Supplementary figure 1.** Surfactant effect on BbLA activity. (A) Effect of 0.05% Triton X-100, Tween 20, Tween 80, Tergitol, CTAB and SDS on BbLA activity. (B) Effect of different Triton X-100 concentrations on BbLA activity.
